# Prognostic and predictive value of human equilibrative nucleoside transporter 1 (hENT1) in extrahepatic cholangiocarcinoma: a translational study

**DOI:** 10.3389/fphar.2023.1274692

**Published:** 2023-10-18

**Authors:** Lenka N. C. Boyd, Lynn E. Nooijen, Mahsoem Ali, Jisce R. Puik, Jasmine Moustaquim, Stephanie M. Fraga Rodrigues, Robert Broos, Ali Belkouz, Laura L. Meijer, Tessa Y. S. Le Large, Joris I. Erdmann, Gerrit K. J. Hooijer, Michal Heger, Hanneke W. M. Van Laarhoven, Eva Roos, Geert Kazemier, Elisa Giovannetti, Joanne Verheij, Heinz-Josef Klümpen

**Affiliations:** ^1^ Department of Surgery, Amsterdam UMC, Location Vrije Universiteit, Amsterdam, Netherlands; ^2^ Department of Medical Oncology, Lab of Medical Oncology, Amsterdam UMC, Location Vrije Universiteit, Amsterdam, Netherlands; ^3^ Cancer Center Amsterdam, Imaging and Biomarkers, Amsterdam, Netherlands; ^4^ Department of Surgery, Amsterdam UMC Location University of Amsterdam, Amsterdam, Netherlands; ^5^ Department of Medical Oncology, Amsterdam UMC Location University of Amsterdam, Amsterdam, Netherlands; ^6^ Department of Surgery, Erasmus MC-University Medical Center, Rotterdam, Netherlands; ^7^ Department of Pathology, Amsterdam UMC Location University of Amsterdam, Amsterdam, Netherlands; ^8^ Jiaxing Key Laboratory for Photonanomedicine and Experimental Therapeutics, Department of Pharmaceutics, College of Medicine, Jiaxing University, Jiaxing, China; ^9^ Department of Pharmaceutics, Utrecht Institute for Pharmaceutical Sciences, Utrecht University, Utrecht, Netherlands; ^10^ Erasmus Medical Center, Laboratory for Experimental Oncology, Department of Pathology, Rotterdam, Netherlands; ^11^ Department of Pathology, Amsterdam UMC Location Vrije Universiteit Amsterdam, Amsterdam, Netherlands; ^12^ Cancer Pharmacology Lab, Associazione Italiana per La Ricerca Sul Cancro (AIRC) Start-Up Unit, Fondazione Pisana per La Scienza, Pisa, Italy

**Keywords:** hENT1, cholangiocarcinoma, perihilar cholangiocarcinoma, distal cholangiocarcinoma, extrahepatic cholangiocarcinoma, prognostic biomarker, predictive biomarker

## Abstract

**Introduction:** Effective (neo) adjuvant chemotherapy for cholangiocarcinoma is lacking due to chemoresistance and the absence of predictive biomarkers. Human equilibrative nucleoside transporter 1 (hENT1) has been described as a potential prognostic and predictive biomarker. In this study, the potential of rabbit-derived (SP120) and murine-derived (10D7G2) antibodies to detect hENT1 expression was compared in tissue samples of patients with extrahepatic cholangiocarcinoma (ECC), and the predictive value of hENT1 was investigated in three ECC cell lines.

**Methods:** Tissues of 71 chemonaïve patients with histological confirmation of ECC were selected and stained with SP120 or 10D7G2 to assess the inter-observer variability for both antibodies and the correlation with overall survival. Concomitantly, gemcitabine sensitivity after hENT1 knockdown was assessed in the ECC cell lines EGI-1, TFK-1, and SK-ChA-1 using sulforhodamine B assays.

**Results:** Scoring immunohistochemistry for hENT1 expression with the use of SP120 antibody resulted in the highest interobserver agreement but did not show a prognostic role of hENT1. However, 10D7G2 showed a prognostic role for hENT1, and a potential predictive role for gemcitabine sensitivity in hENT1 in SK-ChA-1 and TFK-1 cells was found.

**Discussion:** These findings prompt further studies for both preclinical validation of the role of hENT1 and histochemical standardization in cholangiocarcinoma patients treated with gemcitabine-based chemotherapy.

## 1 Introduction

Cholangiocarcinomas are an uncommon collection of tumors arising in the biliary tree, comprising 3% of all gastrointestinal malignancies diagnosed worldwide each year (Banales et al., 2020). The incidence of cholangiocarcinoma is rising and the prognosis remains poor, with a 5-year survival rate of merely 7%–20% (Banales et al., 2020). Based on the anatomical location, cholangiocarcinomas are divided into intrahepatic and extrahepatic cholangiocarcinoma (ECC), of which ECC is the most common, comprising 90% of all diagnosed cholangiocarcinoma (Cillo et al., 2019). ECC is subdivided in perihilar (pCCA) and distal cholangiocarcinoma. Surgical resection remains the only curative treatment for both subtypes (Cillo et al., 2019). However, resection is only possible in 47% of ECC cases due to late presentation, rapid disease progression, and close anatomical location to vital organs (Herman et al., 2014; Kim et al., 2018; Rizvi et al., 2018; Cillo et al., 2019). Even when radical resection is deemed possible, the mean overall 5-year survival rate after resection is only 33% (Nagino et al., 2013; Koerkamp et al., 2015; Kang et al., 2016; Krasnick et al., 2018; Rassam et al., 2018; Regge and Zamboni, 2018). The majority of patients suffering from cholangiocarcinoma present with unresectable, i.e., locally advanced or metastatic, disease (Gaspersz et al., 2018).

Recently, the FDA granted approval for futibatinib as a treatment option for patients with previously treated, unresectable, advanced, or metastatic bile duct cancer who have a specific genetic alteration in fibroblast growth factor receptors (Goyal et al., 2023). However, for the majority of the cholangiocarcinoma patients, palliative chemotherapy by means of gemcitabine-based chemotherapy, often in combination with cisplatin or oxaliplatin, is commonly used as the first-line treatment (Valle et al., 2010). Despite this approach, treatment response varies significantly among ECC patients. The identification of prognostic and/or predictive biomarkers to guide chemotherapy decisions therefore remains an unmet need (Kim et al., 2018). In particular, predictive biomarker discovery and validation and further development towards clinical implementation of these biomarkers is crucial for improvement of effective treatment strategies as well as prevention of unnecessary treatment for ECC patients.

Due to its function and distinct expression pattern, the human equilibrative nucleoside transporter 1 (hENT1) has been widely investigated as a potentially predictive or prognostic biomarker for many tumor types, including cholangiocarcinoma (Randazzo et al., 2020). hENT1 is a transmembrane protein that is expressed in various epithelial cells, including the biliary tract, and is a member of the equilibrative nucleoside transporters family (Pastor-Anglada and Pérez-Torras, 2015). The physiological function of hENT1 is to transport nucleosides and nucleobases so as to maintain cellular nucleoside homeostasis, which is essential for DNA and RNA synthesis and cell proliferation (Young et al., 2008; Boswell-Casteel and Hays, 2017). In addition, hENT1 is the major transmembrane transporter for the intracellular uptake of gemcitabine and capecitabine (Molina-Arcas et al., 2006; Santini et al., 2011). The potential of hENT1 as prognostic biomarker is commonly based on its function as an importer of the nucleosides needed for DNA replication (Boswell-Casteel and Hays, 2017; Isayama et al., 2021), suggesting that lower expression of hENT1 coincides with a worse prognosis. On the other hand, the predictive value of hENT1 is believed to stem from its involvement in the mechanism of action of gemcitabine, a nucleoside analog that disrupts DNA structure and causes double-stranded breaks upon incorporation into DNA (Liu et al., 2017; Roos et al., 2019; Isayama et al., 2021). A higher expression of hENT1 would therefore suggest higher uptake of gemcitabine, potentially leading to increased tumor toxicity and a more beneficial patient outcome (Kim et al., 2018). Nevertheless, existing research investigating the prognostic and predictive significance of hENT1 in cholangiocarcinoma has faced limitations in the form of small sample sizes and the absence of a dependable antibody for precise determination of hENT1 levels by techniques such as Western blot as well as immunohistochemistry. Consequently, conclusive findings regarding the prognostic and predictive value of hENT1 in cholangiocarcinoma are yet to be established (Roos et al., 2019; Isayama et al., 2021).

Accordingly, the aim of this study was to 1) assess which monoclonal antibody is most reliable to determine hENT1 levels in immunohistochemical staining; 2) examine the prognostic value of hENT1 expression in patient-derived tissue samples; 3) investigate the predictive value of hENT1 by assessing sensitivity to gemcitabine before and after specific inhibition of hENT1 in ECC cell lines.

## 2 Materials and methods

### 2.1 Patient characteristics

All consecutive patients diagnosed with pCCA and for which surgical resection was performed in the Amsterdam UMC, location AMC between 1992–2007, and from whom enough tumor tissue was available to build a tissue microarray (TMA), consisting of three cores of 0.6 mm, were included. Baseline characteristics, surgical and pathology data and clinical follow-up were collected and evaluated. Resection margins were defined as R0 when no tumor cells were found on all margins (≥1 mm), R1 when tumor cells were microscopically present in at least one of the resection planes and R2 when macroscopic residual disease was found. The TNM (tumor-node-metastasis status) stage was reassessed for each tumor according to the American Joint Committee of Cancer/Union for International Cancer control, eighth edition. The Institutional Medical Ethics committee of the Amsterdam UMC waived the need for an ethical approval of this study (W20_159).

### 2.2 Tissue microarray construction

Tissue microarray (TMA) was constructed from resected histological tumor tissue, using a TMA instrument (Beecher Instruments, Inc., Silver Springs MD, United States). From each tumor, three cores with a thickness of 0.6 mm were collected from the center of the tumor, to ensure that at least one of the cores would contain tumor cells. Three cores were placed in a recipient block, to exclude effects of heterogeneous antigen expression and cutting or staining artefacts.

### 2.3 Immunohistochemistry

To examine hENT1 expression, immunohistochemistry was performed with two different monoclonal antibodies: one murine-derived (10D7G2) and one rabbit-derived (SP120) antibody. These antibodies were developed and characterized as described previously (Jennings et al., 2001; Mackey et al., 2002; Dabbagh et al., 2003; Spratlin et al., 2004). The SP120 is a commercially available antibody and was obtained from Spring Bioscience Pleasanton, United States. The 10D7G2, a monoclonal antibody that is not commercially available, was obtained from Cross Cancer Institute, University of Alberta in Edmonton, Canada. An overview of the staining process for both monoclonal antibodies can be found in Appendix 1.

### 2.4 Scoring

**FIGURE 1:**
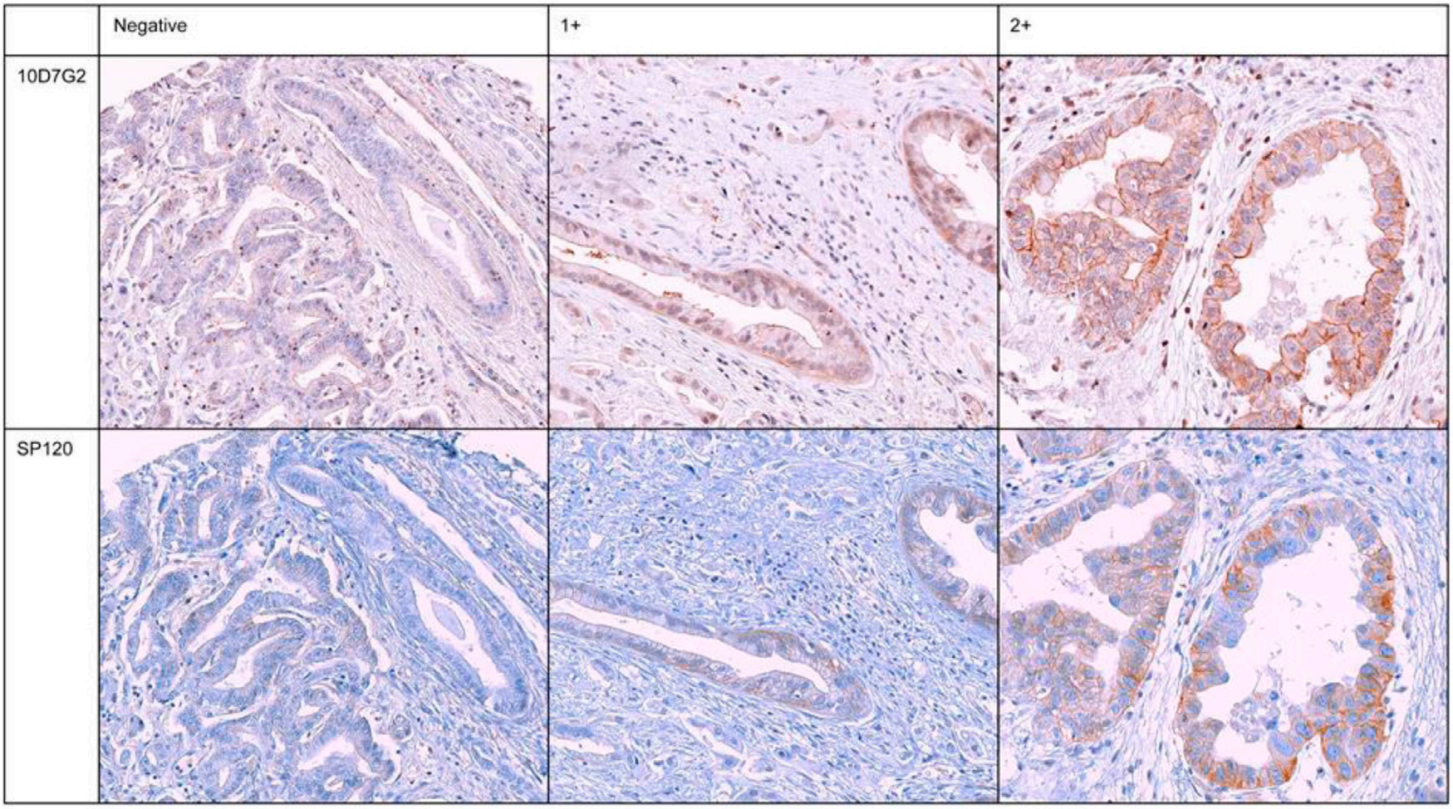
Immunohistochemical staining of six representative and concordant tumour samples for hENT1 expression for both murine-derived (10D7G2) and rabbit-derived (SP120) antibodies. Negative: no staining, 1+: intermediate staining, 2+: strong staining.

The scoring of hENT1 staining was performed by two independent pathologists (ER, GKHJ) in the presence of a researcher (LEN) involved in the present study. To ensure impartiality, the pathologists were blinded to the cases and the cores on the recipient blocks were semi-randomly presented through random allocation. A well-established scoring system, as previously described, was employed to grade the intensity of hENT1 staining in the tumor samples (Spratlin et al., 2004; Santini et al., 2008; Farrell et al., 2009). Staining intensity was graded as absent or negative (0) when there was no staining observed, intermediate (1+) when less than 50% of the cell surface showed positive staining, or strong (2+) when 50% or more of the cell surface showed positive staining, Figure 1. A tumor was considered hENT1 positive, when at least one out of three TMA ECC cores showed positive staining (1+ or 2+). Non-assessable staining was defined as absence of tumor cells in all three TMA cores, inadequate tissue on the TMA due to loss of the tumor tissue during previously performed sectioning resulting in less available cores or if one pathologist scored the sample as positive and the other as negative. As a positive control, staining of the tumor samples was compared to staining of the internal controls (lymphocytes) from the same sample and external controls (Islets of Langerhans) (Spratlin et al., 2004), to verify whether the staining had been precise and specific for hENT1, as hENT1 is normally expressed in the cell membranes of lymphocytes and in the cells of the islets of Langerhans.

### 2.5 Cell culture

Two immortalized distal cholangiocarcinoma cell lines derived from primary tumors, EGI-1 and TFK-1, and the immortalized SK-ChA-1 pCCA cell line, derived from malignant ascites of a patient with extrahepatic cholangiocarcinoma, were selected for this study. The cells were cultured using RPMI 1640 medium supplemented with 2 mM L-glutamine, 10% non-heat inactivated fetal bovine serum (Biowest, Nuaillé, France) and 1% penicillin/streptomycin (Lonza BioWhittaker, Verviers, Belgium). The cells were kept at 37°C and 5% CO_2_ in a humidified incubator. All cell lines were authenticated and tested negative for *mycoplasma* contamination.

### 2.6 siRNA transfection

Cells were seeded in a 96-wells plate and a T75 flask and reverse transfected with hENT1 siRNA inhibitor (Thermo Fisher Scientific, Waltham, MA, United States), at a final concentration of 25 nM siRNA per well, using Lipofectamine RNAiMAX transfection reagent (Thermo Fisher Scientific) according to the manufacturer’s instructions. siRNA control 1 (Silencer^®^ Negative Control #1, ref 4,390,843) and siRNA control 2 (Silencer Negative Control #2, ref 4,390,846) were used as negative control. Cells were collected 24 h and 96 h post-transfection and hENT1 expression was analyzed by Western blot as described in Section 2.7.

### 2.7 Western blot

The basal levels of hENT1 expression and the extent of hENT1 knockdown were assessed using Western blot. Whole-cell lysates were prepared from cells transfected with hENT1 siRNA after 24 h or medium as control, by addition of cell lysis buffer (#9803, Cell Signaling Technology, Danvers, MA, United States) diluted in demineralized water and supplemented with sodium orthovanadate (S6508, Sigma-Aldrich, St. Louis, MO, United States) and protease inhibitor cocktail (11697498001, Sigma-Aldrich), followed by incubation on ice for 15 min. GAPDH was used as internal reference control protein. The samples were centrifuged at 16,000× *g* for 10 min at 4°C. Protein levels in the supernatant were determined with the Bio-Rad protein assay (#500-0006, Bio-Rad Laboratories, Hercules, CA, United States). Finally, 15 μg protein samples were separated on Mini-Protean TGXTM precast gels and transferred to PDVF membranes prior to target detection on the Uvitec gel documentation system (Uvitec Ltd., Cambridge, United Kingdom) using ECLTM Prime Western blotting detection reagent (lot#13601176, GE Healthcare Bio-Sciences, Pittsburgh, PA, United States). Antibodies: the SP120 is a commercially available antibody and was obtained from Spring Bioscience Pleasanton, United States. Antibody binding was detected using enhanced chemoluminescence and densitometric analysis of the images captured on the Uvitec instrument and was performed with ImageJ (U. S. National Institutes of Health, Bethesda, Maryland, United States, https://imagej.nih.gov/ij/).

### 2.8 Cell viability

The sulforhodamine B (SRB) assay was performed to determine the half maximal inhibitory concentration (IC_50_) values for gemcitabine sensitivity for each cell line and their hENT1 knockdown counterpart. Cells were seeded in triplicate in flat bottom 96-well plates (EGI-1, 10.000 cells per well; TFK-1, 10.000 cells per well; SK-ChA-1, 20.000 cells per well) and allowed to attach for 24 h before addition of gemcitabine. A separate control plate was seeded in a similar way and used as a t = 0 plate. Gemcitabine was dissolved in dimethyl sulfoxide (DMSO (Sigma-Aldrich) at a 1-mM stock concentration and stored at −20°C. Titrations of gemcitabine (4—1,500 nM) were added to cells of each cell line. After 72 h, cells were fixed in 5% trichloroacetic acid (Sigma-Aldrich), washed thrice with PBS, stained with 0.4% w/v SRB in 1% acetic acid, and resuspended in 10 mM unbuffered Tris in demineralized water. The absorbance was measured at 490 nm on a BioTek plate reader (BioTek Instruments, Winooski, VT, United States). IC_50_ values were determined using R (R Foundation for Statistical Computing, Indianapolis, IN, United States) and the R-package N-Parameter Logistic Regression (NPLR).

### 2.9 Statistical analysis

Continuous baseline variables were reported as median (interquartile range), and categorical variables as frequencies and percentages. Overall survival (OS) was estimated using the Kaplan-Meier method, and Cox proportional hazards regression was used to examine the association between hENT1 expression and OS.

The proportional hazards assumption was assessed through visual inspection of Schoenfeld residuals and the Grambsch-Therneau test. The functional form of continuous variables was examined using Martingale residuals and nonlinearity was tested by modeling the association between age and overall survival using restricted cubic splines with 3 knots at the 10^th^, 50^th^, and 90^th^ percentile of the variable distribution. (Harrell, 2001). However, as there was no evidence for nonlinearity (nonlinearity test, *p* = 0.78), the restricted cubic spline terms were dropped from the final multivariable Cox regression model.

The IC_50_ values were calculated using 5-parameter logistic regression. To assess whether the IC_50_ was significantly different after transfection with siRNA, the ^10^log-transformed IC_50_ and standard error for the control and treatment condition were used to calculate a relative IC_50_ (i.e., IC_50, siRNA_/IC_50, control_) with confidence intervals and *p* values obtained through Bland and Altman’s method. (Altman and Bland, 2003).

Three sensitivity analyses were performed. First, potential sparse data bias in the multivariable Cox regression model was addressed using Firth’s correction with profile likelihood confidence intervals (Heinze and Schemper, 2001). Second, 3-parameter and 4-parameter logistic regression models were used instead of a 5-parameter logistic regression model to assess whether the estimated difference in IC_50_ between the control and treatment condition was sensitive to the complexity of the model. Third, a proportional odds ordinal regression model was used instead of linear regression to assess differences in IC_50_ between the control and treatment condition, as the proportional odds model is more robust to the distribution of the continuous outcome variable (Harrell, 2001).

The inter-observer variability was assessed by calculating Cohen’s kappa. Only tumor samples with similar scoring results by both pathologists were included for survival analysis. The OS was defined as the time from the date of surgery to the date of death or last follow-up. Survival status was verified with the Dutch municipal registry. Survival curves were compared using the log-rank test and the reverse Kaplan-Meier method was used to calculate median follow-up.

A two-sided *p*-value lower than 0.05 was considered to indicate statistical significance. All statistical analyses were performed in R, version 4.2.1 (R Foundation for Statistical Computing).

## 3 Results

### 3.1 Patient characteristics

Tissue samples of 71 patients with ECC (i.e., pCCA) who underwent resection were stained to assess hENT1 expression. Of these, 41 patients underwent a major hepatectomy which included a resection of the extrahepatic bile ducts, 19 patients underwent a hilar resection which included the extrahepatic bile ducts, and 11 patients underwent a resection of the extrahepatic bile ducts only (Table 1). Among these patients, 41 (58%) were male and median age at diagnosis was 62 (interquartile range, 53–68) years. The majority of patients were classified as T2a (n = 34, 48%), 17 patients (24%) had lymph node metastasis (N1: n = 15/N2: n = 2) while one patient had developed metastasis in the liver, which was discovered perioperatively. A negative (R0) resection margin was observed in 32 patients (45%) and the majority of patients (n = 27, 38%) exhibited poorly differentiated tumors (G3, n = 27, 38%). Thirty-nine (55%) patients developed tumor recurrence during follow-up, and ten patients were subsequently treated with palliative chemotherapy.

**TABLE 1:** Patient characteristics.

Characteristic		
Age at surgery, median (IQR)	62	(53—68)
Female (%)	41	(58%)
Tumor size and extent		
T1	16	(23%)
T2a	34	(48%)
T2b	14	(20%)
T3	2	(3%)
T4	5	(7%)
Regional lymph node involvement (N*)		
N0	45	(63%)
N1	15	(21%)
N2	2	(3%)
Nx	9	(13%)
Distant metastases (M*)		
M0	70	(99%)
M1	1	(1%)
Differentiation grade		
Grade 1: well differentiated	11	(16%)
Grade 2: moderately differentiated	24	(34%)
Grade 3: poorly differentiated	27	(38%)
Not available	9	(13%)
Resection margin R)		
R0	32	(45%)
R1	30	(42%)
R2	9	(13%)

### 3.2 Detection of hENT1 expression with SP120 results in higher inter-observer agreement

Tumor samples of all 71 patients were evaluated for hENT1 by immunohistochemistry with both the rabbit-derived (SP120) and murine-derived (10D7G2) antibodies (Figure 1). When assessing hENT1 expression using the SP120 antibody, 43 (61%) tumor samples were found to be hENT1 negative and 11 (16%) samples were found positive for hENT1 expression. However, hENT1 expression was not assessable in 17 samples using SP120 (24%). Overall, when using SP120, from the 11 positively scored samples, seven showed intermediate staining (1+), two showed strong (2+) staining, two samples were either scored as highly positive (2+) or intermediate positive (1+). The Cohen’s kappa for the two pathologists using the SP120 was 0.85 (standard error, 0.06), indicating a high inter-observer agreement. When assessing hENT1 expression using the 10D7G2 antibody, 21 (30%) tumor samples were found to be negative for hENT1 expression and 16 (23%) tumor samples were found positive for hENT1 expression. hENT1 expression was not assessable for 34 tumor samples (48%). The Cohen’s kappa for the two pathologists using the 10D7G2 was 0.49 (standard error, 0.12), indicating a moderate inter-observer agreement. An overview of all staining results for both SP120 and 10D7G2 is shown in Table 2.

**TABLE 2:** Interobserver variability in hENT1 expression assessment.

	Pathologist 1	Pathologist 2	Overall
hENT1 expression SP120			
Positive	11 (16%)	15 (21%)	11 (16%)
Negative	47 (66%)	43 (61%)	43 (61%)
Not assessable	13 (18%)	13 (18%)	17 (24%)
hENT1 expression 10D7G2			
Positive	19 (27%)	26 (37%)	16 (23%)
Negative	31 (44%)	24 (34%)	21 (30%)
Not assessable	21 (30%)	21 (30%)	34 (48%)

hENT1 = human equilibrative nucleoside transporter 1, SP120 = rabbit-derived monoclonal antibody, 10D7G2 = murine-derived monoclonal antibody.

In total, 9 out of 25 (36%) samples classified as hENT1 negative according to the SP120 antibody, were classified as hENT1 positive according to the 10D7G2 antibody. In addition, 9 out of 14 (64%) classified as hENT1 positive according to the 10D7G2 antibody were classified as hENT1 negative according to the SP120 antibody.

### 3.3 Overall survival was different using the SP120 *versus* 10D7G2 antibody for hENT1

**FIGURE 2:**
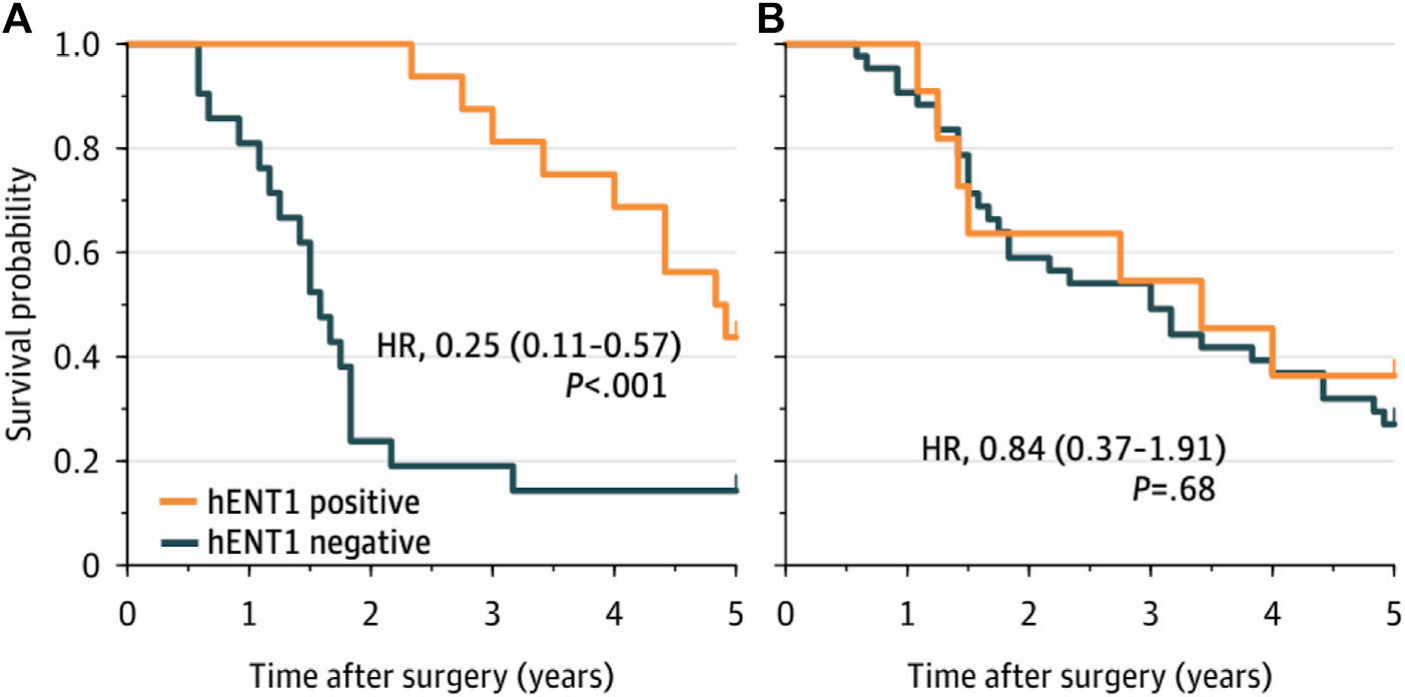
Overall survival for patients with hENT1 positive and negative ECC, as determined by the 10D7G2 antibody **(A)** and the SP120 antibody **(B)**. hENT1 positive was defined as a sample that was classified as positive by both pathologists, hENT1 negative was defined as a sample that was classified as negative by both pathologists.

The median follow-up was 20 years (95% confidence interval [CI], 16—24 years). The median, 5- and 10-year OS of the complete cohort was 41 months (95% CI 27.5—54.5), 36% and 7%, respectively. Using the SP120 antibody, the median OS was 41 months (95% CI 8.6—73.4 months) in patients with positive staining for hENT1 and 36 months (95% CI 21.1—50.9 months) in patients with negative staining (*p* = 0.780). Using the 10D7G2 antibody, the median OS was 58 months (95% CI 46.2—69.8 months) in patients with positive staining for hENT1 compared to 19 months (95% CI 15.6—22.4 months) in patients with negative staining (*p* < 0.001) (Figure 2).

### 3.4 Decreased gemcitabine sensitivity after transfection with hENT1 inhibitor

**FIGURE 3:**
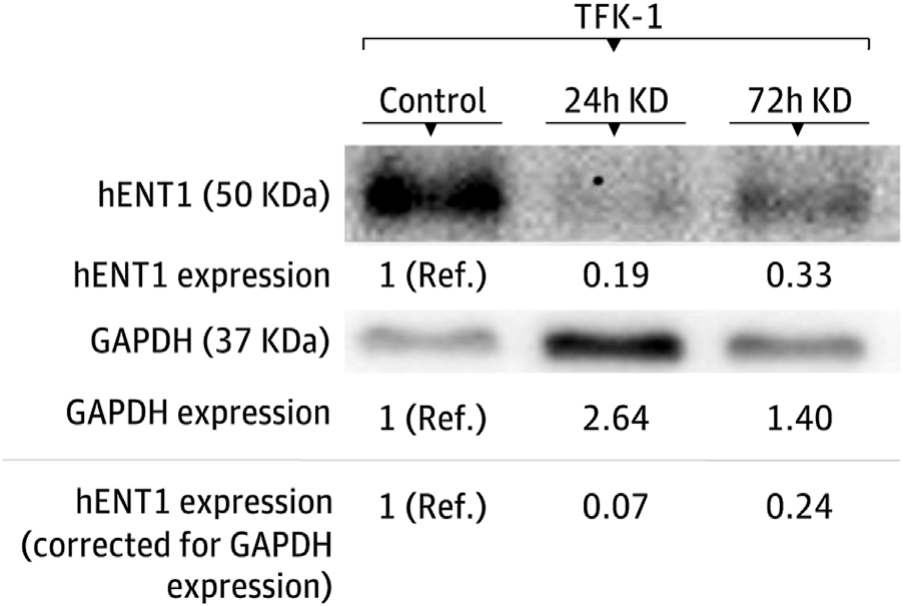
Modulation of hENT1 expression in the TFK-1 cell line. Representative Western blots of hENT1 and GAPDH protein expression. The expression in the control condition is used as the reference group. KD denotes siRNA-mediated hENT1 inhibition, Ref. indicates the reference condition.

Sensitivity to gemcitabine before and after hENT1 inhibition was investigated using the ECC cells EGI-1, TFK-1, and SK-ChA-1. In all these models, a decrease in hENT1 expression was observed after transfection with the hENT1 siRNA. In particular, the expression of hENT1 was studied at the protein level, using Western blotting and densitometry (Figure 3 and Supplementary Figure S1).

**FIGURE 4:**
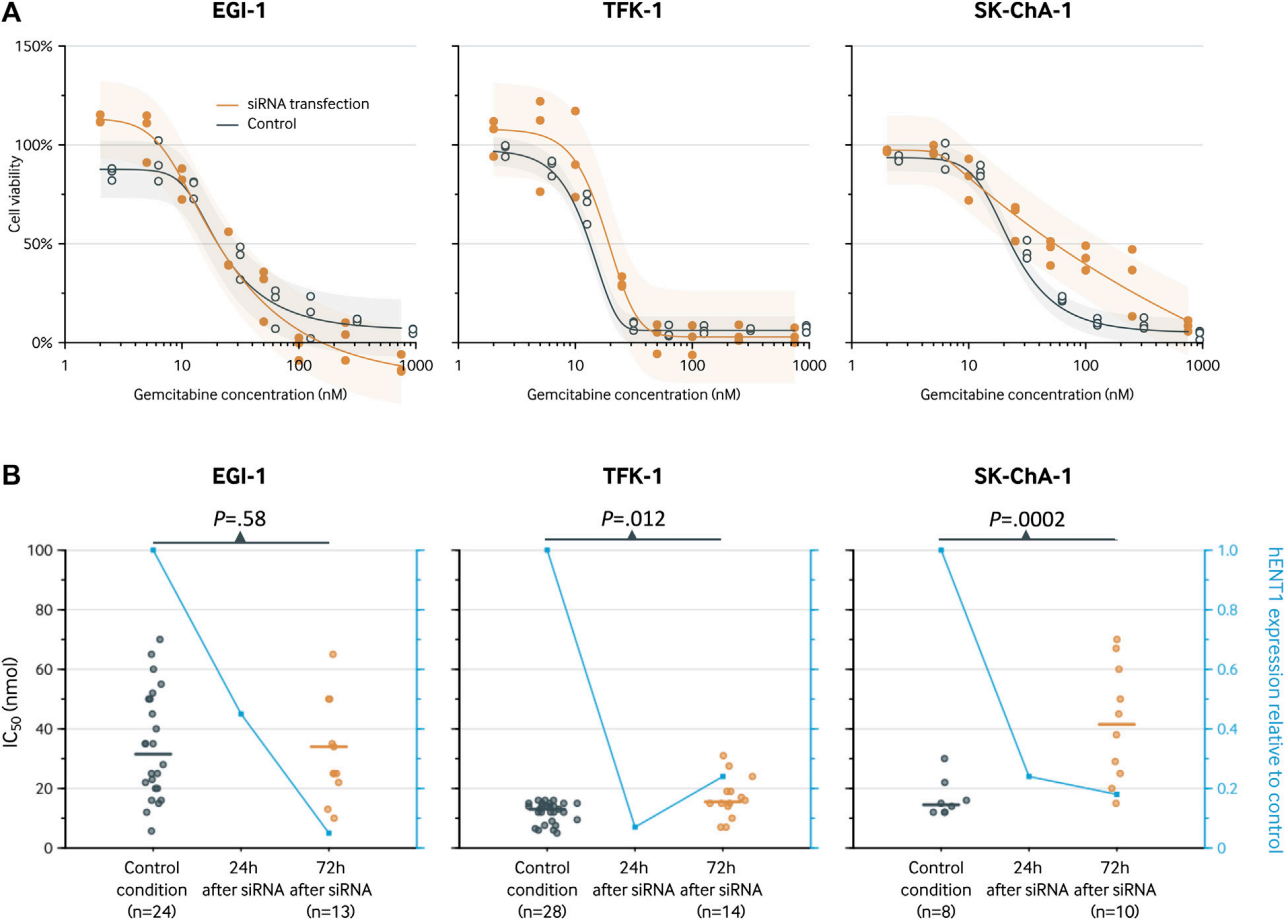
Analysis of the hENT1 expression and of the chemosensitivity to gemcitabine (IC50 after 72-h treatment) in ECC cells. **(A)**, cell viability curve for gemcitabine before and after transfection with siRNA. Results are shown for a 5-parameter logistic regression model based on three replicates for each gemcitabine concentration. The following concentrations were used: 2, 5, 10, 25, 50, 100, 250, and 750 nM. The estimates for the siRNA knockdown group and control group are slightly staggered horizontally to prevent visual overlap. **(B)**, IC_50_ (left *y*-axis) for the control condition and after 72 h. The blue line represents the hENT1 expression relative to the control condition (right *y*-axis).

After siRNA-mediated inhibition of hENT1, the cell viability in the EGI-1 cell line was similar to the control condition with an IC_50_ of 20 nM (95% CI, 16–27 nM; Figure 4) *versus* 20 nM (95% CI, 14–31 nM; *p* = 0.99), respectively. In contrast, the IC_50_ was significantly higher after siRNA transfection in both the TFK-1 cell line (IC_50_, 19 vs*.* 13 nM; relative IC_50_, 1.46 [1.14 to 1.86]; *p* = 0.003) and the SK-ChA-1 cell culture (IC_50_, 54 vs*.* 23 nM; relative IC_50_, 2.38 [1.10 to 5.15]; *p* = 0.028). These estimates did not markedly change when using a 3- and 4-parameter logistic regression model instead of a 5-parameter logistic regression model (Supplementary Table S1 and Supplementary Figures S1, S2), indicating that the relative IC_50_ values were not influenced by the number of parameters in the regression model.

## 4 Discussion

This study shows that the two most commonly used antibodies for hENT1 are either unreliable due to substantial interobserver variability (10D7G2) or show no prognostic value in ECC (SP120), both in unadjusted and adjusted analyses. However, *in vitro* studies indicated a predictive value of hENT1 in ECC cell lines. Specifically, gemcitabine sensitivity decreased after inhibition of hENT1, both in the main regression analysis and when using alternative models.

The current study is the first to compare the two most commonly used antibodies for hENT1 expression in tissue of patients with resected ECC or other types of biliary tract cancer not receiving adjuvant chemotherapy. Previous research has focused on the prognostic and predictive value of SP120 and 10D7G2 in pancreatic ductal adenocarcinoma (PDAC) (Randazzo et al., 2020), in which similar estimates of inter-observer agreement were observed for SP120 (Cohen’s kappa, 0.89 and 0.78). However, in contrast to our results, these previous studies did show an association between SP120 staining and OS (Randazzo et al., 2020). It is unclear whether this discrepancy is related to histopathological differences between pancreatic ductal adenocarcinoma and cholangiocarcinoma, or due to technical or methodological differences. Adherence to best practices for immunohistochemical staining could help standardize the methodology of different studies, and allow more reliable comparisons across studies. In addition, we observed a large discrepancy in antibody staining, as a high percentage of samples classified as positive by one antibody was classified as negative by the other antibody. Similar findings have been described in a previous systematic review of hENT1 antibodies for hepatobiliary cancers, although a definite explanation for the discordance in results between hENT1 antibodies is lacking (Vos et al., 2019).

Although there are other methods to examine hENT1 expression, immunohistochemistry has several advantages, as it is relatively inexpensive, fast, and routinely available. However, the potential of immunohistochemistry is limited by intra-tumoral heterogeneity of hENT1 expression and temporal changes in hENT1 expression. Specifically, the presence of heterogeneity in hENT1 expression within a tumor can pose challenges when assessing hENT1 expression through immunohistochemistry on single biopsy samples, as the results may not accurately represent the overall hENT1 expression profile of the entire tumor (Walter et al., 2017). Hence, it is advisable for future research to investigate the significance of intra-tumoral heterogeneity and the evolution of such heterogeneity over time by examining multiple biopsies at various stages, including diagnosis, post-resection, and in cases of recurrence. This approach would provide a comprehensive understanding of how intra-tumoral heterogeneity develops and its potential therapeutic implications.

Our results in the ECC cells are in line with previous studies examining the predictive value of hENT1 for gemcitabine response in other solid tumors, including non-small-cell lung carcinoma (NSCLC) and PDAC. Specifically, *in vitro* studies have shown a significant correlation between basal expression levels of hENT1 and gemcitabine IC50 values in twenty-two NSCLC cell lines, and the use of the nucleoside transport inhibitors nitrobenzylmercaptopurine riboside and dipyridamole resulted in significantly reduced sensitivity to gemcitabine (Achiwa et al., 2004). Similar results were observed with dipyridamole and nitrobenzylthioinosine in three pancreatic cancer cell lines, indicating the active involvement of hENT1 in gemcitabine uptake and its influence on the antiproliferative effect of this drug (Giovannetti et al., 2006).

However, there is a lack of previous data specifically reported for ECC cells, and the findings regarding intrahepatic CCA cell lines have been contradictory. One study on gemcitabine-resistant variants of human intrahepatic CCA cell lines, KKU-M139 and KKU-M214, showed significantly lower expression levels of hENT1 mRNA in the gemcitabine-resistant cells compared to their parental cells (Wattanawongdon et al., 2015). However, this study did not investigate the effects of hENT1 inhibition, and the resistant cells exhibited modulation of other key determinants of gemcitabine activity, such as upregulation of the ribonucleotide reductase subunit M1 (target of gemcitabine) and downregulation of deoxycytidine kinase, the enzyme catalyzing the limiting step for the activation of gemcitabine. Additionally, both gemcitabine-resistant CCA cells showed increased expression of PKC, as well as phosphorylation of FAK and ERK1/2, indicating the involvement of multiple signaling pathways in the chemoresistant behavior of these models. Conversely, a more recent functional analysis of intrahepatic CCA cell lines revealed that silencing hENT1 inhibited cell proliferation and induced apoptosis in HUH-28 cells that expressed hENT1 on the cell membrane, but not in SNU-1079 cells where the transporter was only present in the cytoplasm. These findings were correlated with clinical data, demonstrating that membrane hENT1 was associated with proliferation and worse survival in resected intrahepatic CCA patients. However, these patients did not receive adjuvant treatments. In contrast, our meta-analysis of immunohistochemical biomarkers in patients with biliary tract cancers treated with gemcitabine-based chemotherapy, including 26 studies involving 1,348 patients with resected or advanced biliary tract cancers, showed the opposite trend: higher levels of hENT1 expression were associated with longer OS and disease-free/progression-free survival (Belkouz et al., 2019).

Although a decrease in gemcitabine sensitivity was observed after siRNA-mediated inhibition of hENT1 in the TFK-1 and SK-ChA-1 cell lines, there was no apparent difference in gemcitabine sensitivity in the EGI-1 cell line. This finding might be related to differences in KRAS mutation status between cell lines. Specifically, EGI-1 cells harbor heterozygous KRAS mutations, whereas TFK-1 and SK-ChA-1 lack KRAS mutations (Yeung et al., 2017). As mutant KRAS can interfere with siRNA-mediated gene silencing in pancreatic cancer cells, the presence of KRAS mutations in EGI-1 cell lines could have resulted in impaired siRNA-mediated gene silencing. The mechanism behind impaired siRNA-based treatment efficacy in the presence of KRAS mutations could be related to KRAS mutations promoting cellular resistance to siRNA by modulating key components of the RNAi machinery, such as Dicer and Ago2, as reported previously (Hsu et al., 2005).

Accurate and reproducible prognostic and predictive biomarkers are necessary to inform personalized shared decision making, select the optimal therapy for individual patients, and improve care of patients with ECC. Combining immunohistochemical hENT1 expression with several routinely measured biomarkers, e.g., CA19-9, could improve the predictive value and facilitate the implementation of a cost-effective model to estimate treatment effects. However, alternative methods to determine hENT1 expression using, e.g., RNA sequencing or PCR techniques, could potentially overcome the lack of robust antibodies for hENT1 protein detection. For instance, an RNA sequencing approach has recently been used in a comprehensive biomarker study that examined the predictive value of hENT1 mRNA in a cohort of advanced PDAC patients that received either modified FOLFIRINOX or gemcitabine/nab-paclitaxel (Hsu et al., 2005). However, RNA sequencing and PCR techniques require more thorough laboratory expertise and, compared with immunohistochemistry, may be less feasible to implement in the clinic.

This study has several strengths and limitations. First, we explored the prognostic and predictive value of hENT1 using both clinical and experimental data to explore the biological mechanism underpinning the association between gemcitabine sensitivity and hENT1. Second, we assessed the robustness of our results using sensitivity analyses, in which we changed modeling assumptions. These analyses yielded consistent results with our primary findings. However, it is important to acknowledge the retrospective nature of the study and the relatively limited sample size, despite this being the largest cohort of ECC patients examined for this particular biomarker (Belkouz et al., 2019). As a result, the precise prognostic value of hENT1 cannot be definitively determined, highlighting the necessity of establishing standardized methodology before utilizing hENT1 status as a predictive biomarker in clinical practice. These findings also underscore the importance of incorporating additional tissues and circulating biomarkers, within larger prospective trials, as well as conducting further preclinical investigations to address the uncertainties surrounding the clinical significance of hENT1 in ECC.

## 5 Conclusion

The present findings indicate a predictive role of hENT1 for gemcitabine sensitivity *in vitro*, and highlight the importance of further immunohistochemical standardization and preclinical validation of the role of hENT1 in cholangiocarcinoma.

## Data Availability

The original contributions presented in the study are included in the article/Supplementary Material, further inquiries can be directed to the corresponding author.
