# Supplementary material for: Prognostic and predictive value of human equilibrative nucleoside transporter 1 (hENT1) in extrahepatic cholangiocarcinoma: a translational study
Source: Front Pharmacol. 2023 Oct 18;14:1274692. doi: 10.3389/fphar.2023.1274692 (PMC10619907; doi:10.3389/fphar.2023.1274692)
Supplement: Supplementary file 1 [file DataSheet1.docx]

**Table 1. Relative IC_50_ in sensitivity analyses.**

|  | **IC_50_ control**  **(95% CI)** | | **IC_50_ siRNA knockdown**  **(95% CI)** | | | **Relative IC_50_**  **(95% CI)** | | **P value** |
| --- | --- | --- | --- | --- | --- | --- | --- | --- |
| **EGI-1** |  |  |  |  | |  |  |  |
| 5-parameter model | 20 | (16 to 27) | 20 | (14 to 30) | 1.00 | | (0.62 to 1.59) | 0.99 |
| 4-parameter model | 22 | (17 to 28) | 22 | (16 to 33) | 1.01 | | (0.64 to 1.57) | 0.98 |
| 3-parameter model | 22 | (15 to 34) | 21 | (15 to 31) | 0.94 | | (0.53 to 1.65) | 0.83 |
| **TFK-1** |  |  |  |  |  | |  |  |
| 5-parameter model | 13 | (12 to 14) | 19 | (15 to 24) | 1.46 | | (1.15 to 1.85) | 0.002 |
| 4-parameter model | 12 | (11 to 13) | 19 | (15 to 24) | 1.53 | | (1.20 to 1.94) | 0.0007 |
| 3-parameter model | 13 | (11 to 15) | 19 | (15 to 25) | 1.47 | | (1.12 to 1.94) | 0.006 |
| **SK-ChA** |  |  |  |  |  | |  |  |
| 5-parameter model | 23 | (21 to 25) | 54 | (26 to 122) | 2.38 | | (1.09 to 5.17) | 0.029 |
| 4-parameter model | 25 | (22 to 28) | 58 | (26 to 128) | 2.36 | | (1.07 to 5.23) | 0.034 |
| 3-parameter model | 25 | (21 to 31) | 56 | (27 to 124) | 2.22 | | (1.02 to 4.84) | 0.046 |

**Supplementary figure 1. Modulation of hENT1 expression.** Representative Western blots of hENT1 and GAPDH protein expression. For each cell line, the expression in the control condition is used as the reference group. KD denotes siRNA-mediated hENT1 inhibition, Ref. indicates the reference condition.


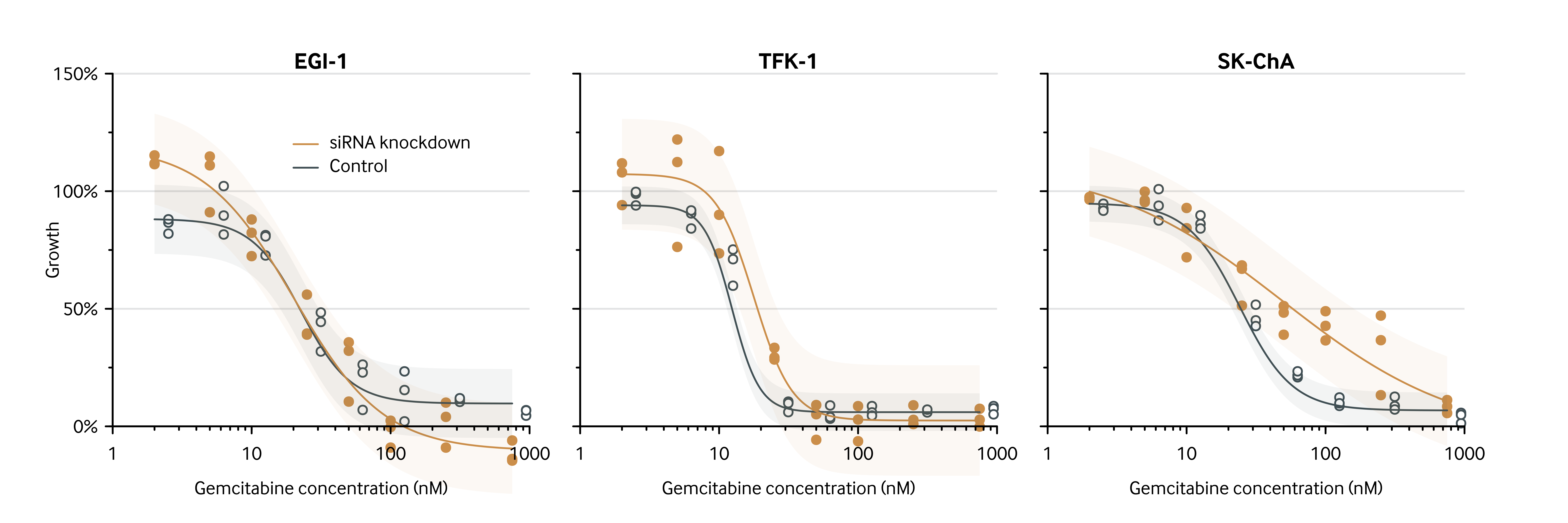


**Supplementary Figure 2. Dose-response curve of gemcitabine with the 4-parameter model.**

**
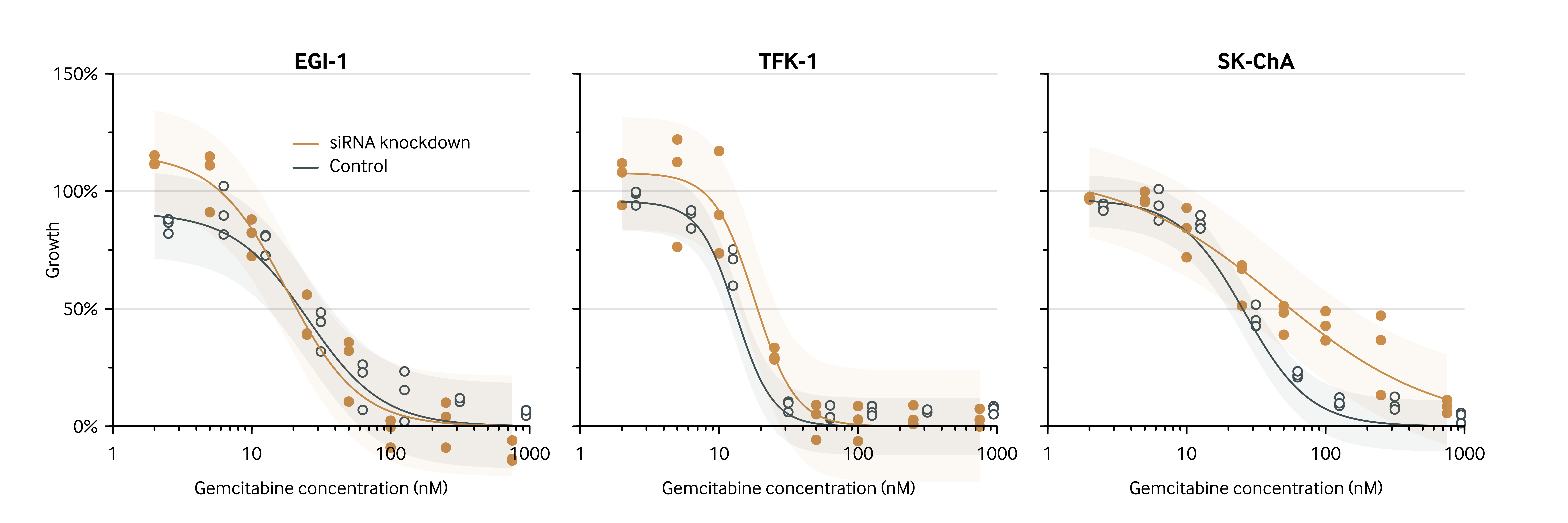
**

**Supplementary Figure 3. Dose-response curve of gemcitabine with the 3-parameter model.**

**
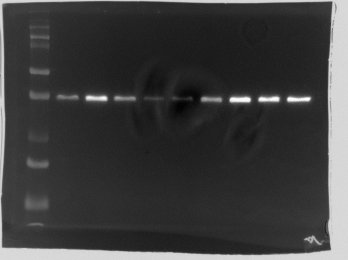
**

**Supplementary Figure 4. Full, uncut western blot for GAPDH.**

**
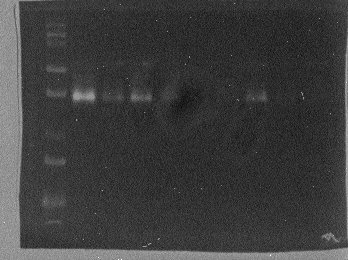
**

**Supplementary Figure 5. Full, uncut western blot for hENT1.**

Appendix

For staining with the 10D7G2 antibody, all formalin-fixed, paraffin-embedded tumor TMA sections were deparaffinized in xylene baths and hydrated gradually through decreasing concentrations of ethanol. To remove endogenous peroxidase activity, sections were incubated in 0.3% hydrogen peroxidase solution in methanol for 20 minutes. After rinsing in distilled water, the sections were placed in a Tris-EDTA antigen-retrieval solution (10mM Tris Base, 1mM EDTA solution, 0.05% Tween 20, pH 9.0) for 20 minutes at 120°C in a pressure cooker. Tumor sections were then washed in Phosphate-Buffered Saline with Tween (PBS-T) and incubated overnight at 4°C with hENT1 mouse monoclonal antibody (10D7G2) with a dilution of 1:10. Next, sections were washed in PBS-T and incubated with secondary goat HRP-Polymer anti-mouse antibody (Immunologic) for 30 minutes at room temperature, followed by rinsing in PBS-T. Bound antibody was detected using Bright DAB+ detection kit (Immunologic) and the DAB signal was enhanced by soaking the slides in 1% CuSO4 in 0.9% NaCl for 5 minutes. Subsequently, slides were counterstained with 1:5 Hematoxylin (Klinipath), dehydrated through graded alcohol and cleared in Xylene, and finally mounted with Pertex.

Immunohistochemical staining for the rabbit monoclonal antibody (SP120) was performed using an automated slide preparation system (Benchmark XT, Ventana Medical Systems, Tucson Arizona, USA). Antigen retrieval was performed with CC1 standard and the sections were incubated in a 1:150 antibody dilution for 32 minutes at 37°C. The signal detection for immunohistochemistry was performed with a biotine free ultraview universal DAB detection Kit (Ventana medical systems). Finally, the slides were dehydrated through graded alcohol, cleared in Xylene and mounted with Pertex.
